# Mcl-1 and tumor cell persistence

**DOI:** 10.18632/oncotarget.3035

**Published:** 2014-12-26

**Authors:** Lukas W. Pfannenstiel, Brian R. Gastman

**Affiliations:** Lerner Research Institute Department of Immunology, Dermatology and Plastic Surgery Institute, Taussig Cancer Center, and Head and Neck Institute, Cleveland Clinic, Cleveland, OH

Effective genotoxic cancer therapies induce not only classical apoptotic cell death, but also senescence, a persistent state of cell cycle arrest accompanied by distinct morphological and biochemical changes. A number of studies in both animal and human models demonstrate that senescence plays a key role in limiting tumor growth and correlate with favorable clinical responses and prognosis [1,2]. As a result, the induction of senescence is now recognized as a primary target for a number of established chemotherapy drugs and experimental therapies [3].

Tumor suppressor genes (TSGs) like p53 and Rb are major upstream inducers of senescence in response to a variety of stresses, including chemotherapy and oncogenic activation. Consequently, the functional losses of TSGs are key events in the process of oncogenesis. Chemotherapy induced senescence, however, can occur even in the absence of TSGs, adding to the complexity of senescence induction. When and how chemotherapy-induced senescence creates a true permanent growth arrest and not merely quiescence (a reversible state) remains controversial. Clinically, chemotherapy can induce histologic findings of senescence that can be correlated with treatment success in many cancer types. However, these lines of therapies are also observed to arrest tumor growth for a period of time, only to later give way to rapid cancer proliferation similar to a quiescent state giving rise to a period of adaptation.

Mcl-1 and Bcl-xL are pro-survival proteins of the Bcl-2 family and are well-known for their anti-apoptotic activity via their shared Bcl-2 homology (BH) binding domains. Mcl-1 and Bcl-xL are highly conserved, and the loci containing these genes are amplified in a wide variety of human cancers [4]. Recent studies have also found a novel anti-senescence activity unique to Mcl-1 that appears to be mediated by a domain outside of the BH binding pocket [5].

A recent study by Barbara Jonchére and colleagues further explores the relationship between Mcl-1/Bcl-xL expression and apparently reversible forms of chemotherapy-induced senescence in colorectal cancer (CRC) cells [6]. Building upon their previous work on oncogenic-RAS mediated senescence in HCT116 and LS174T CRC cells, the authors induced senescence in these cells by treatment with the active metabolite of irinotecan, a topoisomerase I inhibitor used as a frontline therapeutic for colorectal cancer. After treatment, both cell lines underwent senescence. Subsequent culture of treated cells in colony assays revealed a small population of cells that persisted through irinotecan treatment and resumed growth. Though these persistent cells were still largely senescent *in vitro* based on standard assays, they readily formed xenograft tumors in mice with growth kinetics identical to untreated cells. Interestingly, persistent LS174T cells appeared more transformed and invasive than untreated cells in that they contained a large percentage of cells with polyploid DNA content and readily grew in low-adherence soft-agar and matrigel culture assays. Persistent cells were determined to not express any known colorectal cancer stem cell or cancer initiating cell (CIC) markers, and were not sensitive to treatment with salinomycin, a compound known to be specifically toxic to colorectal CIC.

Building on their work with oncogene induced senescence, Jonchére *et al*. evaluated the expression of both Bcl-xL and Mcl-1 in this model. Both proteins were found to be selectively enhanced in these irinotecan-treated, persistent cells. Knockdown of Mcl-1 by siRNA and the inhibition of Bcl-xL by chemical inhibitor (ABT-737) significantly increased cell death and decreased the number of persistent cells. Perhaps most intriguingly, persistent LS174T cells were found to consist of two populations based on apparent senescent state: a more “senescent” population that demonstrated higher β-galactosidase activity and p21 expression; and a population that more closely resembled dividing cells with less senescence markers, and greater proliferative capacity in normal clonogenic assays. Contrary to appearances, however, it was the more “senescent” population that demonstrated higher Mcl-1 and Bcl-xL expression, as well as significantly greater growth in soft agar and matrigel assays.

The mechanism by which the heterogeneity that arises in LS174T cells during chemotherapy treatment remains undefined. It is clear, however, that this heterogeneity can affect the sensitivity of individual cells to treatment-induced senescence and apoptosis. Although this finding is consistent with the classic observation that senescent cells are relatively resistant to apoptotic signals, it is novel in that they can later proliferate in such an aggressive manner. It is not surprising that Mcl-1 and Bcl-xL were found to be significant in the development of persistence after drug treatment as both are known to contribute to resistance to drug treatment [7]. Failure of irinotecan treatment in colorectal cancer is a well-known clinical phenomenon, and the study by Jonchére *et al*. suggests that development of more efficacious treatment regimens can still include the induction of senescence but would have to address the development of small populations of persistent cells through targeting Bcl-2 family members, like Bcl-xL and Mcl-1. This study adds to the growing acceptance amongst clinicians and scientists that multiple simultaneous combination therapies will be required to enhance multiple forms of cellular demise in order to maximize clinical outcome.
